# Supplementary material for: Mechanisms governing the pioneering and redistribution capabilities of the non-classical pioneer PU.1
Source: Nat Commun. 2020 Jan 21;11:402. doi: 10.1038/s41467-019-13960-2 (PMC6972792; doi:10.1038/s41467-019-13960-2)
Supplement: Supplementary file 7 — Source data [file 41467_2019_13960_MOESM7_ESM.zip › Source_Data/FigureS5/FigureS5B_MotifsETS1specificPeaks/homerResults/motif3.similar.html]

motif3

## Information for motif3

C
T
G
A
C
T
G
A
C
G
T
A
A
G
T
C
A
C
G
T
C
G
T
A
A
G
T
C
C
T
G
A
  
Reverse Opposite:  

A
G
C
T
A
C
T
G
A
C
G
T
C
G
T
A
A
C
T
G
A
C
G
T
A
G
C
T
A
G
C
T
  

|  |  |
| --- | --- |
| p-value: | 1e-126 |
| log p-value: | -2.915e+02 |
| Information Content per bp: | 1.899 |
| Number of Target Sequences with motif | 321.0 |
| Percentage of Target Sequences with motif | 28.28% |
| Number of Background Sequences with motif | 352.9 |
| Percentage of Background Sequences with motif | 4.32% |
| Average Position of motif in Targets | 102.3 +/- 47.6bp |
| Average Position of motif in Background | 103.0 +/- 73.4bp |
| Strand Bias (log2 ratio + to - strand density) | 0.1 |
| Multiplicity (# of sites on avg that occur together) | 1.32 |
| Motif File: | file (matrix) reverse opposite |

### Similar de novo motifs found

|  |  |  |  |  |  |  |  |
| --- | --- | --- | --- | --- | --- | --- | --- |
| Rank | Match Score | Redundant Motif | P-value | log P-value | % of Targets | % of Background | Motif file |
| 1 | 0.833 | C T A G C T A G G C T A A G T C A C G T T G C A A G T C T G C A G C A T | 1e-111 | -255.843187 | 29.96% | 5.97% | motif file (matrix) |
| 2 | 0.868 | C G A T A G C T A C T G A C G T C G T A A C T G A C G T | 1e-110 | -253.826069 | 25.64% | 4.08% | motif file (matrix) |
| 3 | 0.817 | C T A G C T A G C G T A A G T C A C G T C G T A A G T C | 1e-102 | -237.041719 | 32.16% | 7.65% | motif file (matrix) |
| 4 | 0.781 | C T G A C T A G C T G A C G T A T A G C G A C T C T G A A G T C G T C A G C T A G C A T | 1e-49 | -113.903543 | 11.28% | 1.62% | motif file (matrix) |
